# The first protocol for assessing the welfare of dromedary camels (*Camelus dromedarius*) kept under nomadic pastoralism

**DOI:** 10.3389/fvets.2024.1416714

**Published:** 2024-06-11

**Authors:** Barbara Padalino, Laura Menchetti

**Affiliations:** ^1^Department of Agricultural and Food Sciences, University of Bologna, Bologna, Italy; ^2^School of Environmental and Rural Science, Faculty Science, Agriculture, Business and Law, University of New England, Armidale, NSW, Australia; ^3^School of Biosciences and Veterinary Medicine, University of Camerino, Camerino, Italy

**Keywords:** animal welfare, feeding, housing, health, behavior, camels, compound welfare indices

## Abstract

There is no protocol to measure the welfare level of dromedary camels (*Camelus dromedarious*) kept under pastoralism—the predominant husbandry system of this species. This study therefore aimed to develop and describe a protocol for measuring welfare levels in dromedary camels kept under nomadic pastoralist conditions—. The indicators for each welfare principle (i.e., Good Feeding, Good Housing, Good Health, and Appropriate Behavior) were tailored to the specific conditions of camel pastoralism, drawing from the currently available protocol for assessing welfare in dromedary camels kept in intensive and semi-intensive systems. This adaptation was achieved using a structured literature search and Expert Knowledge Elicitation (EKE). The developed protocol, covering animal-, resource-, and management-based indicators, comprises two assessment levels: ‘Caretaker-Herd level’ and ‘Animal level’. The Caretaker-Herd level is a face-to-face interview of about 10 min including 16 questions, spit into the four welfare principles, and a visual observation of applied animal handling practices. The ‘Animal level’ encompasses a behavioral observation and a visual clinical inspection of randomly selected individual dromedary camels, about 5 min/camel. The ‘Animal level’ includes 27 welfare indicators displayed for each welfare principle. The present study also includes the score for each indicator, the model for aggregating indicators’ scores into compound indices for each welfare principle (PAI), and how to classify the herds based on the PAIs or to produce an overall welfare index for each herd. Even if the proposed protocol needs to be applied, refined, and validated, it is a first step toward a standardized method to collect data related to dromedary camel welfare kept under pastoralism. This framework may ultimately guide herd managers, animal health practitioners, experienced advisers, and lawmakers in fostering optimal conditions and proposing welfare standards for dromedary camels in pastoralist settings.

## 1 Introduction

Large camelids or Old World camels (*Camelus dromedarius*), dromedary or one-humped camel, and Bactrian or two-humped camel) are known for their resilience in arid and semi-arid landscapes. The assumed camel’s adaptive resilience in hostile settings may keep the notion of ‘animal well-being’ disregarded and considered independent of human influence. In recent decades, however, the global population of large camelids has surged due to factors like climate change-induced desertification and the demand for sustainable meat and milk production (1). This shift has led to significant changes in camel breeding practices (1, 2). Concretely, growing social and economic interests in dromedary camel husbandry at intensive and semi-intensive production systems have significantly appeared on the scene during the past three decades (3). This situation has parallelly promoted an increase in the scientific actions that are implemented, and which deal with almost any discipline applied to this animal species (1). Nonetheless, applied scientific studies on the effects of different housing systems and handling practices, on camel behavior, health, and welfare are scarce (4–7). Hence, both the traditional and currently changing dynamics of camel breeding necessitate an objective assessment of their impact on the camel’s well-being for their long-term sustainability.

A pioneer protocol for the assessment of welfare in dromedary camels has been recently set up by Padalino and Menchetti (8). It applies to dromedary camels kept at intensive and semi-intensive housing systems and develops a model considering overall welfare indices (9). This protocol involves a combination of indicators, evaluated at three levels, namely Caretaker-level, Herd-level, and Animal-level. These indicators align with animal welfare principles (‘Good Feeding,’ ‘Good Housing,’ ‘Good Health,’ and ‘Appropriate Behavior’) based on the Welfare Quality^®^ and European Animal Welfare Indicators (AWIN) projects (10, 11). In addition, to include indicators of positive welfare, in the protocol also the Five Domains model was considered (12). However, this protocol cannot be applied to dromedary camels kept under pastoralism, as in these pastoralist nomadic environments, animal husbandry methods still notably diverge from those employed in modern semi-intensive and intensive camel farming systems.

The majority of dromedary camels are raised under nomadic pastoralist conditions in the arid and semi-arid ecosystems of Africa and Asia (13, 14). Pastoralism involves the practice of raising livestock for subsistence, with practices varying from agropastoralism (a blend of plant cultivation and herding) to predominantly herding animals (15). Concretely, dromedary camel pastoralism holds profound social and economic significance for local human livelihoods. Socially, it forms the backbone of many communities, shaping cultural practices and traditions. The communal nature of camel herding often strengthens social bonds, as communities collaborate in managing herds and sharing resources. Economically, camel pastoralism provides a sustainable source of income through the sale of camel milk, meat, hides, and wool. Camels’ ability to thrive in harsh environments makes them invaluable also for transportation and agricultural activities, enhancing productivity and enabling communities to access remote markets (16, 17). Dromedary camels raised in pastoralist conditions, as more aligned with their natural behaviors, have not raised, up to date, many welfare concerns from the community and the policy-makers (7). However, like all the animals kept in extensive systems, they can face other several challenges that can influence their homeostasis and thus impact both production and welfare (18). Dromedary camels kept in these semi-arid regions deal with unpredictable rainfall cycles, alternating between dry and rainy seasons. Forage availability is inconsistent, leading to periods of hunger during the dry season and potential starvation in droughts. Limited access to drinking water in arid areas also forces dromedaries to endure thirst, especially during the dry season when watering points are distant. Night enclosures, if present, are basic, exposing the animals to thermal and predator stresses (7). Furthermore, remote nomadic pastoral areas often lack veterinary services and essential drugs, resulting in regular occurrences of diseases, parasites, and associated pain and distress (19). However, neither empirical data nor a tool to objectively assess and score the welfare status of dromedaries reared in pastoralist nomadic environments exist up to the present.

To fill this gap of knowledge, this study aimed to develop and describe a novel protocol for assessing welfare in adult dromedary camels reared in pastoralist nomadic environments. Welfare principles and indicators were adapted from the existing protocol for assessing welfare in dromedary camels raised in intensive and semi-intensive systems (8) to suit the unique conditions of camel pastoralism.

## 2 Methods

### 2.1 Selection of welfare indicators

The currently available protocol for assessing the welfare of dromedary camels kept at intensive and semi-intensive systems (8) was used as the starting point. However, the nomadic nature of dromedary camel pastoralism poses a challenge. These animals typically do not inhabit a stationary pen but roam across varied landscapes even within the same day in response to the caretaker’s strategic decisions mostly regarding foraging locations and water sources. Hence, to effectuate this adaptation, a comprehensive literature search and a series of meetings, during which several unstructured Expert Knowledge Elicitations (EKEs) (20) were conducted.

In particular, a scientific literature search was conducted using the following keywords: ‘camel welfare’, ‘camel feeding, ‘camel behavior’, ‘camel housing’, ‘camel health’, ‘camel management’, ‘camel reproduction’, and ‘camel pastoralism’. ScienceDirect, PubMed, and Scopus were the research databases used for tracking recent academic publications. It is noteworthy that the literature review was confined to English-language publications and the last 20 years. Briefly, 7,589 peer-reviewed papers (i.e., 7,456 research studies, 116 reviews, and 17 book chapters) were retrieved and the authors screened them, excluding those that were not related to animal welfare indicators applied under an extensive husbandry system. Given the scarcity of species-specific literature, the researchers also took into account peer-reviewed papers on other species, with particular emphasis on those aimed to develop and apply welfare protocols in horses and ruminants that are kept in groups and in extensive husbandry systems (21, 22).

During the meetings, a small group of experts (*n* = 10), selected from the authors’ network (see acknowledges for details on them) based on their experience in camel behavior and animal welfare, discussed and developed the recording sheets using EKEs. Starting from the recording sheets published and applied previously for the assessment of the welfare of dromedary camels kept under intensive and semi-intensive systems (8, 9), different animal-, resource-, and management-based indicators were kept or adapted in accordance with prevalent practices in the literature (23–25). While the previous protocol for dromedary camels kept in intensive systems (8) has three levels of investigation, only two levels of investigations, namely ‘Caretaker-Herd level’ and ‘Animal level’, were agreed upon for the current protocol. For each level of investigation, the experts agreed on a variety of management- resource- and animal-based welfare indicators for each welfare principle (‘Good Feeding’, ‘Good Housing’, ‘Good Health, and ‘Appropriate Behavior’) according to the previous protocol (8) and the Welfare Quality^®^ and AWIN methods (10, 11). However, as in the previous protocol (8), indicators more related to a positive welfare state were also selected and included. The discerning selection of welfare indicators adhered to the tenets of reliability (capability to yield consistent outcomes across various time points or when conducted by different assessors) and feasibility (time and cost efficiency), as expounded in the scientific literature for diverse species (26, 27). For instance, indicators necessitating extensive laboratory analyses, such as metabolic profiling, were not included to ensure feasibility. Additionally, considerations of animal welfare and operator safety led to the exclusion of invasive indicators or those requiring physical contact, acknowledging the potential stress these procedures could induce in untamed camels. Animal-based indicators are predominant at the ‘Animal level’, whereas the ‘Caretaker-Herd level’ primarily encompasses resource- and management-based indicators. The developed protocol, including the Caretaker-Herd level and Animal level recording sheets, was then piloted on a small number of herds (*n* = 5) on a total of 60 animals in Egypt to refine the questions and to test the reliability and feasibility of the chosen indicators.

### 2.2 Scoring of indicators, model for aggregation of indicators’ measures, and system for classification of herds

Starting from the scoring systems proposed by Menchetti et al. (9) a 3-step process was retained useful for this protocol to aggregate the scores of each welfare indicator in each principle and assessment level into compound indices. The two systems for herd categorization proposed by the previous paper (9) (i.e., one based on the score profiles of the partial indices of the welfare principles and one on the categorization into tertiles of the total score; see paragraph 3.4) were also discussed and agreed upon using EKEs.

## 3 Results

**Figure 1:**
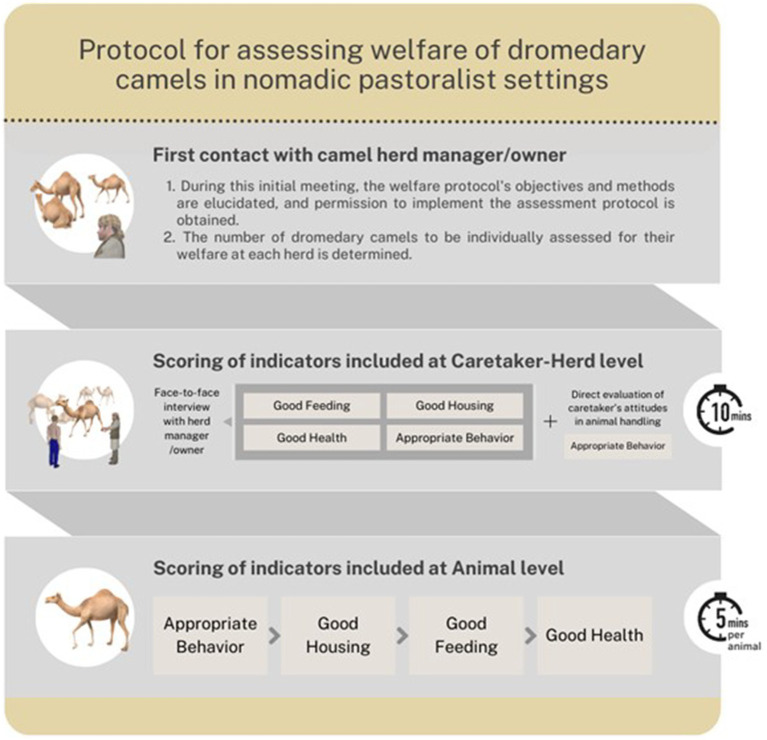
Graphical representation of the sequential data collection and processing during the on-field application of the protocol to assess the welfare of dromedary camels in nomadic pastoralist settings.

The logical sequence for on-field data collection is detailed in Figure 1. Prior to conducting assessment activities, assessors are required to undergo standardized training that comprehensively covers the entire protocol. This training should include the description and application of each indicator, the order for data collection, possible constraints in protocol application, and adherence to sanitary rules.

The assessment must be scheduled to align it with pastoralists’ routines. If the assessor cannot communicate in the same language, a native speaker must contact the herd manager/camel herd owner and plan a meeting to conduct the welfare assessment. During this meeting, the welfare protocol’s objectives and methods must be elucidated, and permission to implement the assessment protocol must be obtained. It is crucial to clarify that the welfare assessment poses no risk to the involved dromedaries and caretakers, and all procedures are non-invasive. To minimize those risks, the assessment of dromedary camels should occur from a distance to ensure the animals are unaware of or undisturbed by the assessor’s presence, the dromedary camels should only be approached gently, and the assessment must be stopped if a camel displays behavior that poses a danger to people or the animal itself. Assessors should keep this conversation concise to prevent subjective influence on results (28).

To ascertain the number of dromedary camels for assessment, it is imperative to first identify and discard those animals that are suffering from severe or acute pathological processes and are under treatment. After that, the minimum number of animals to be randomly selected for assessment at each herd can be calculated, taking into account the number of adult animals (i.e., > 3 years old) that are present within the herd and adhering to the AWIN’s guidelines for the selection of small ruminants (28), and as already reported in the other protocol (8) (Table 1).

**Table 1:** Rule of thumb to determine the minimum number of dromedary camels to be individually assessed for their welfare at each herd.

Number of adult dromedary camels in the herd	Minimum number of dromedary camels to be individually assessed for their welfare
<15	All animals
15–19	13
20–24	16
25–29	19
30–34	21
35–39	24
40–44	26
45–49	28
50–59	29
60–69	32
70–79	35
80–89	37
90–99	39
100–124	41
125–149	44

### 3.1 Dromedary camel welfare assessment at the caretaker and herd levels

Table 2 shows the recording sheet at the Caretaker-Herd level. The assessment at the Caretaker-Herd level includes a face-to-face interview with the herd manager/owner (i.e., the person in charge of the management of the herd under pastoralism conditions). The face-to-face interview is specifically composed of 16 close-ended queries that explore various aspects, including feeding, watering and health practices, housing conditions, and the human-animal relationship. As a part of the assessment at this level, the assessors must also observe from a distance when caretakers (s; i.e., the person involved mainly in the handling, feeding, and watering practices of the animals) are handling the camels. The assessors should focus on the attitudes and manners of the caretakers, and whether some equipment (e.g., stick) is used and the manner it is used, aligning with the principles outlined in the OIE Terrestrial Animal Health Code (42). Assessment at the ‘Caretaker-Herd level’ should take approximately 10 min.

**Table 2:** Recording sheet to use during the dromedary camel welfare assessment at the Caretaker-Herd level.

Day:___, Time:____	Location	Season	Temperature:Humidity	Lux
Principle	Question/welfare indicator	Answer/observation	Scoring scale	Notes
Good feeding	How often do you feed the camels?	Grazing for around 10–12 h per day + supplementation	0	
		Only grazing for 10–12 h per day	1	
		Only grazing for less than 6–8 h per day	2	
	How often do you water the camels?	Always available	0	
		Available more than once daily	1	
		Available less than once daily	2	
	*Total Observed Score for Good Feeding at Caretaker-Herd level*			
Good housing (Environment)	Do camels have a resting place overnight?	Yes	0	
		No	2	
	How many adult animals do you have in your herd?^1,2^	<30 camels (*Small size*)	0	
		>30 camels (*Large size*)	2	
	Do the camels have access to shaded areas?	Free access during the whole day	0	
		For a short period of time per day	1	
		Never	2	
	Do you practice any type of predator control?^3^	Yes	0	
		No	2	
	*Total Observed Score for Good Housing at Caretaker-Herd level*			
Good health	Who routinely assesses the camel’s health?	A veterinarian	0	
		A non-veterinarian	1	
		Not conducted	2	
	Who treats the camels when they are sick?	A veterinarian	0	
		A non-veterinarian	1	
		Not conducted	2	
	Are vaccinations routinely conducted?	Yes	0	
		No	2	
	Is deworming routinely conducted?	Yes	0	
		No	2	
		A non-veterinarian	1	
		Not conducted	2	
	What is the 1-year-old calf mortality rate?^4,5,6,7,8,10,11^	Below 10%	0	
		Over 10%	1	
		Records not available^9^	2	
	Do you identify your animals?	Yes, using non-invasive methods	0	
		Yes, using pain-induced practices	1	
		No^9^	2	
	Do your animals have the possibility to contact with other livestock herds (commingling)?	No	0	
		Ratherly	1	
		Yes	2	
	*Total Observed Score for Good Health at Caretaker-Herd level*			
Appropriate behavior	Do you have any aggressive/dangerous animals in your herd?	No	0	
		Yes, but only during the breeding season	1	
		Yes	2	
	How many years of experience in handling camels do you have?	More than 10	0	
		Between 5 and 10	1	
		< 5 years	2	
	What is the ratio between number of caretakers and number of animals kept at the herd?	Ratio ≥ 0.05	0	
		Ratio < 0.05	2	
	Caretaker attitudes in animal handling^12,13^	Speaks, touch and/or whistles softly/quietly	0	
		Speaks, touch and/or whistles harshly/loudly	1	
		Speaking/shouting impatiently, forceful use of stick/hand	2	
	*Total Observed Score for Appropriate Behavior at Caretaker-Herd level*			

Questions are split accordingly with each welfare principle and possible answers are scored on a three-point scale where 0 is the best welfare condition. ^1^Benaissa, Mimoune (29); ^2^Schulte and Klingel (30); ^3^Farah, Nyariki (31); ^4^Kaufmann (32); ^5^Nagy, Reiczigel (33); ^6^Megersa, Regassa (34); ^7^Wernery (35); ^8^Jaji, Elelu (36); ^9^Greene (37); ^10^Nagy, Skidmore and Juhasz (38); ^11^Ihuthia (39); ^12^Waiblinger, Menke and Coleman (40); ^13^Hultgren, Wiberg (41).

The recording sheet (Table 2) shows also the scoring for each possible answer, in line with the literature (9). Briefly, a 0–2 scale was used, namely 0 = good welfare, 1 = compromised welfare, and 2 = low welfare. In the case of welfare indicators represented as a binary response (e.g., presence/absence), only scores of 0 (good welfare) and 2 (low welfare) are considered, to keep the same weight for each indicator and in agreement with the previous scoring systems (9, 10).

As in the previous protocol (8), before starting the interview (Table 2), the assessor should record the environmental parameters (at least air temperature and relative humidity) using a weather station, and the lux with a lux meter. On the recording sheet, the assessor should also note down the date, time of the day, season, and location. In regards to the location, it is important to understand whether the animals have been there for some days or are just in transit.

### 3.2 Dromedary camel welfare assessment at the animal level

Table 3 shows the recording sheet for the assessment at the Animal level. The assessment of each individual dromedary camel should take approximately 5 min per each dromedary.

**Table 3:** Recording sheet to use during the dromedary camel welfare assessment at the Animal-level.

Principle	Welfare indicator	Observation	Scoring scale	Note
Good feeding	Food availability	Yes, and of good quality	0	
		Yes, but of low quality	1	
		No	2	
	Water availability	Yes, fresh and clean water is available	0	
		Yes, but of low quality (e.g., dirty, warm)	1	
		No	2	
	Body Condition Score (BCS)	BCS = 3 (good body condition)	0	
		BCS = 2 or BCS = 4 (Moderate body condition)	1	
		BCS = 0–1 or BCS = 5 (cachexia or obesity)	2	
	*Total Observed Score for Good Feeding at Animal level*			
Good housing (Environment)	Currently available shade	Yes	0	
		No	2	
	Risk of injury/foreign body (e.g., presence of rubbish and other foreign objects which could be eaten or could injury the camel)	No	0	
		Yes	2	
	Presence of ectoparasites	No	0	
		Yes	2	
	Camel coat cleanliness	Clean	0	
		Partially clean	1	
		Dirty	2	
	Tethered	No	0	
		Yes	2	
	Restrained into two/three legs	No	0	
		Yes	2	
	Hobbled	No	0	
		Yes	2	
	Voluntary resting behavior	Yes	0	
		No	2	
	*Total Observed Score for Good Housing at Animal level*			
Good health	Presence of bleeding	No	0	
		Yes	2	
	Presence of injury (open wounds)	No	0	
		Yes	2	
	Presence of swollen joints	No	0	
		Yes	2	
	Presence of lameness	No	0	
		Yes	2	
	Presence of skin disorders	No	0	
		Yes	2	
	Presence of discharge (nose, eye, vulva)	No	0	
		Yes	2	
	Presence of diarrhea	No	0	
		Yes	2	
	Presence of respiratory disorders	No	0	
		Yes	2	
	Presence of other health disorders*	No	0	
		Yes	2	
	Presence of pain-induced management practices (cauterization, branding, nose pag, mutilation)	No	0	
		Yes	2	
	Evident pain	No	0	
		Yes	2	
	*Total Observed Score for Good Health at Animal level*			
Appropriate behavior	Positive social camel-camel interactions (cow-calf contact, allogrooming, sniffing)	Yes	0	
		No	2	
	Aggressive camel-camel interactions	No	0	
		Yes	2	
	Stereotypies	No	0	
		Yes	2	
	Feeding or rumination	Yes	0	
		No	2	
	Approaching test	Positive	0	
		Neutral	1	
		Negative	2	
	*Total Observed Score for Appropriate Behavior at Animal level*			

Indicators are split accordingly with each welfare principle and possible answers are scored on a three-point scale where 0 is the best welfare condition. *Please write down the specific health disorders observed.

Concerning the flow of steps at this assessment level, after having randomly chosen the animal to assess, a 3-min behavioral observation must be conducted without disturbing the camel. During this observation, the assessors must note down the indicators included in the Good Housing (i.e., access to shaded areas, risk injury and foreign bodies, and voluntary resting behavior), Good Feeding (i.e., food and water availability), and Appropriate Behavior (i.e., positive and aggressive camel-camel interactions, stereotypy, feeding and rumination). After the behavioral observation, the assessor must conduct the approaching test as in the other protocol (8). Briefly, the assessor approaches the camel gradually from the side, taking one step at a time to minimize stress and extending his/her arm and hand in a non-threatening manner. The test stops if the camel exhibits avoidance or aggressive behavior or when the tester successfully approaches and places a hand close to the camel’s shoulder. Three different behavioral responses can be observed. Negative responses encompass defensive, anxious, avoidant, or aggressive behavior. A neutral response is characterized by the camel remaining calm and relaxed, paying no further attention to the test person. On the other hand, a positive response involves the camel approaching the test person with a positive interest, engaging in sniffing, and allowing touch or petting by the test person.

After this behavioral test, a thorough visual clinical inspection of the camel is carried out to determine its Body Condition Score (BCS) and the rest of the indicators of Good Housing (presence of ectoparasites, cleanliness, and physical restrain), as well as to check for clinical signs, presence of pain-induced practices, and injuries listed in the Good Health principle (Table 3). Body condition must be assessed using the 0–5 validate scale based on visual examination of the camel’s ribs, ischial and coxal tuberosities, the hollow of the flank, and the recto-genital zone (43), and then scored on a three-point scale, considering both cachexia and obesity as a welfare concern (8). Concerning the presence of bleeding and open wounds (both shallow and deep wounds), it was agreed after the piloting that bleeding refers to the visible flow of blood from an injury or wound, whereas open wounds refer to injuries where the skin is compromised, exposing underlying tissues and not necessarily resulting in bleeding, and old scars, where the skin was not compromised anymore, must not be considered as wounds. In addition, to properly score the presence or absence of lameness, if the dromedary camel has remained in a resting position during the application of the protocol, it has to be asked, in a gentle manner, to stand up and walk for a few steps at the end of the assessment. This way, the camel’s gait can be evaluated to determine if the animal can bear weight wholly or evenly, and if the course of movement is disturbed or not. By contrast, if the animal can only stand up with help or not at all and cannot bear weight on one leg or shows a relieving posture, assessing the camel in motion will not be necessary to confirm the presence of lameness.

At the end of the examination, the assessor should give his/her impression of whether the camel is in pain or not (‘Evident pain’; Table 3). Currently, there are no sensitive scales for recognizing and scoring pain through physiological and behavioral responses in dromedary camels, but a composite pain scale based on the literature available on other mammal species has been recently proposed (44) and could be used as a possible reference.

Before moving to the assessment of a second animal, it is also suggested to mark the assessed one, to avoid reassessing it, as being free to move, it would be hard to track and recognize the assessed animals. It may be useful to have one of the caretakers available in case of the need to calm down a camel or for a more specific veterinary inspection in case of the identification of a possible disease that needs a more invasive diagnostic.

The recording sheet at the Animal level (Table 3) shows also the scoring for each possible answer; the scale used to score the indicators gathered at this assessment level is identical to the scale previously defined at the Caretaker-Herd level.

### 3.3 Model for aggregation of measures from welfare indicators

**Figure 2:**
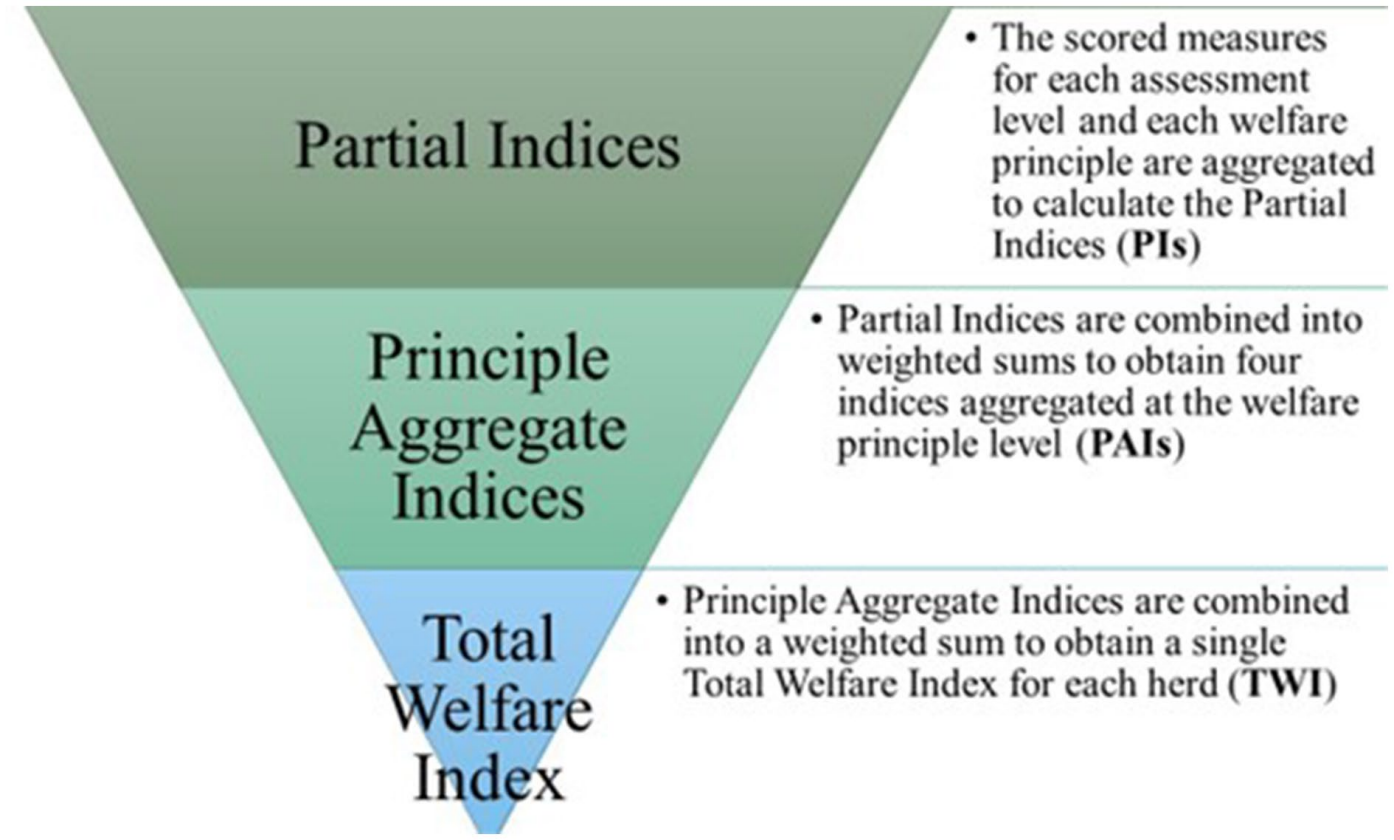
The three-step process of aggregation of measures from indicators collected to assess the welfare of dromedary camels kept under nomadic pastoralism conditions.

Figure 2 shows the 3-step process of aggregation of measures from welfare indicators applied to the current assessment protocol in line with the literature (9).

In the first step, scores are converted into partial indices (PIs). A total of 8 PIs per herd are calculated, namely: Good Feeding at the Caretaker-Herd level, Good Housing at the Caretaker-Herd level, Good Health at the Caretaker-Herd level, Appropriate Behavior at the Caretaker-Herd level, Good Feeding at the Animal level, Good Housing at the Animal level, Good Health at the Animal level, and Appropriate Behavior at the Animal level. In the calculation of PIs, the original 0–2 scale is transformed into a 0–100 scale, where 0 represents the lowest (i.e., unacceptable welfare) value and 100 the highest (i.e., optimal welfare). PIs are computed for each assessment level (*i*) and each principle (*j*) using the following formula:


$${\mathrm{PI}}_{i,j}=100-\left(\frac{\sum_{m=1}^{n_{i,j}}{\left(\mathrm{Score}\;\mathrm{of}\;\mathrm{welfare}\;\mathrm{indicator}\right)}_{m}x\;100}{k_{i,j}}\right)$$

where *i* is the assessment level *i*, *j* corresponds to the principle level *j*, n refers to the number of welfare indicators included in the *j* principle of the *i* level, and k is the highest possible total score of each principle *j* within each assessment level *i*.

The second step involves combining PIs into weighted sums, resulting in indices aggregated at each welfare principle (Principle Aggregate Indices or PAIs). Four different aggregate indices are obtained per herd: Good Feeding Index, Good Housing Index, Good Health Index, and Appropriate Behavior Index. These PAIs range from 0 (worst condition/unacceptable welfare) to 100 (best condition/optimal welfare) and offer an overall assessment for each welfare principle by herd including the scores obtained at the two levels of investigation. The relative weight of each level of assessment within the calculation of the PAIs was determined regarding the quality of the information provided by each of them. Specifically, a lower weight (20%) was attributable to the PIs of ‘Caretaker-Herd level’ given the fact that the recording sheet designed for this level of assessment primarily scores resource- and management-based indicators based on the responses provided by the caretaker, hence subjected to potential ‘questionnaire bias’ (45). The PAI for each principle *j* is calculated as follows:


$${PAI}_{j}=\left({\mathrm{PI}}_{\mathrm{Caretaker}-\mathrm{Herd},j}\;x\;0.20\right)+\left({\mathrm{PI}}_{\mathrm{Animal},j}\;x\;0.80\right)$$

where *j* corresponds to the principle level *j*.

During the third and last step, PAIs are further combined to derive the Total Welfare Index (TWI) for each herd. The TWI, representing the overall assessment regardless of assessment level and welfare principle, ranges from 0 (poor welfare) to 100 (optimal welfare). All PAIs are combined with equal weights (25%) to calculate the TWI as follows:


$$\begin{array}{l} TWI=\left(\mathrm{Good}\;\mathrm{Feeding}\;\mathrm{Index}\;x\;0.25\right)+\\ \;\left(\mathrm{Good}\;\mathrm{Housing}\;\mathrm{Index}\;x\;0.25\right)+\\ \;\left(\mathrm{Good}\;\mathrm{Health}\;\mathrm{Index}\;x\;0.25\right)+\\ \;\left(\mathrm{Appropriate}\;\mathrm{Behavior}\;\mathrm{Index}\;x\;0.25\right) \end{array}$$

While interpreting both PAIs and TWI, the assessors must take into account the location, the season and the environmental conditions (i.e., T and H), and whether reschedule other assessments during other climatic conditions and/or in other locations.

### 3.4 Criteria and welfare classes for the classification of the herds

Different welfare classes were delineated to classify the herds based on the scores of the four Principle Aggregate Indices (PAIs) as suggested by the Welfare Quality^®^ Network (46, 47) and already applied in the protocol to assess the welfare of dromedary camels kept in intensive systems (9) (Table 4).

**Table 4:** Quantitative criteria and respective welfare categories for the classification of camel herds based on Principle Aggregate Indices (PAIs).

Parameter	Criteria	Welfare category
Principle Aggregate Indices (PAIs)	>60 for each PAI and > 80 for at least two PAIs	Excellent
	>30 for each PAI and > 60 for at least three PAIs	Satisfactory
	>20 for each PAI and > 30 for at least three PAIs	Unsatisfactory
	Failure to meet the abovementioned criteria	Unacceptable

**Figure 3:**
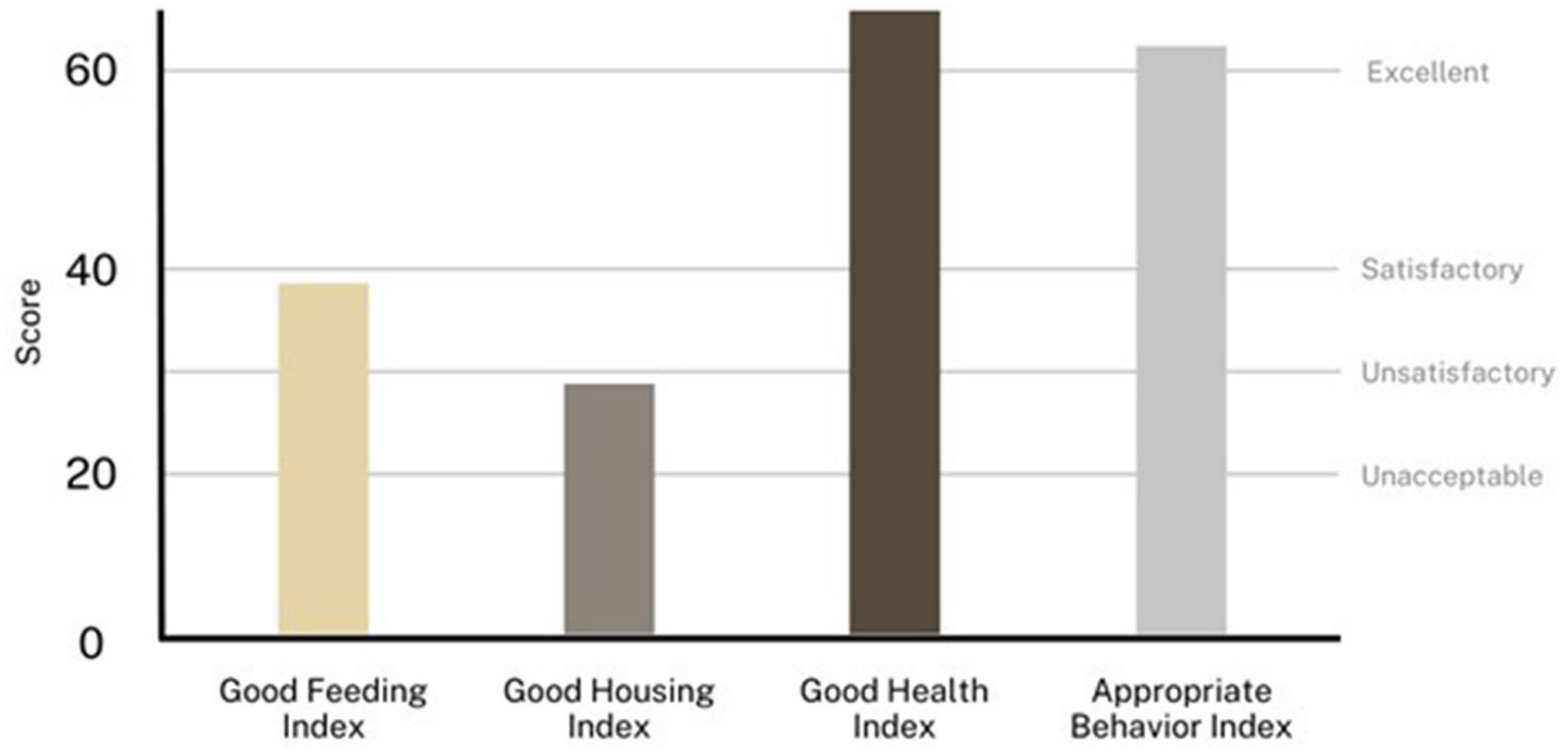
Example of classification of a herd having a score of 38 for the Good Feeding Index, 28 for the Good Housing Index, 65 for the Good Health Index, and 62 for the Appropriate Behavior Index. This herd is classified as Unsatisfactory, as all PAIs are above 20 but only three PAIs are above 30.

An example of possible graphic results of the classification of the herds based on the PAIs is reported in Figure 3. This classification is useful as it is easy to identify the welfare principles where the herds have some issues, so recommendations to enhance them can be suggested considering the welfare indicators that were inappropriate in that particular welfare principle.

Another classification of the herds can be performed using the TWI and statistical binning. Specifically, three classes can be established based on TWI tertiles, and they are labeled using a “traffic light” system: “green light” if the pen’s TWI falls within the third tertile, “orange light” if it is in the second tertile, and “red light” if it is in the first tertile (9). However, to apply the binning method, the protocol needs to be applied to a specific population to accurately calculate the tertiles and compare the herds of the assessed population among them.

## 4 Discussion

This study developed and presented an original protocol to assess the welfare of adult dromedary camels, in any physiological states and during any seasons, kept under nomadic pastoralist conditions. This protocol was developed adapting the currently accessible protocol for assessing the welfare of dromedaries reared in intensive and semi-intensive systems (8, 9), using the literature and structured EKE. The main adaptation from the previous protocol was to eliminate a level of investigation, namely, the Herd level, where the majority of the parameters were on the pen where the animals were kept, which clearly is lacking under pastoralism. The recording sheet at the Caretaker and Herd levels were therefore joined. This integration captures the direct interactions between the caretaker(s) and the camels (i.e., animal handling and care practices) and the impact of the environmental factors that inherently shape the caretaker’s decisions in regard to herds’ transhumance, thus the overall well-being of the herd (48). The Caretaker-Herd level of assessment has been adapted from previously developed surveys focused on how camel caretakers manage their herds (49) and their proficiency in collecting long-term data of sufficient quality (32). Additionally, information regarding the effect of group size (29, 30), control of potential predators (31), the absence of an animal identification/traceability program (37), stock person actions, and caretaker/camel ratio (40, 41, 50) and calf mortality rate (33, 39) was retrieved and adapted from the specific literature. This conglomerate of information serves the purpose of investigating aspects of the management that are hard to capture with the instantaneous evaluation at the Animal level, considering that these animals keep moving based on the caretaker’s decisions. The caretaker information is therefore crucial to evaluate the level of the welfare of the animals kept under that particular management. Moreover, information on the general management of the animals also helps to define longer-term challenges and opportunities for animals, in agreement with the most modern concepts of welfare (such as those of balance between positive and poor welfare) which emphasize the need to take into consideration the cumulative experiences over time (51). However, the data collected as a questionnaire are clearly reported information, so subject to bias and, although they assume a limited weight in the calculation of total welfare (i.e., 20%), must be reported as a possible limitation of the current protocol (9). Overall, the proposed protocol is a first step in the evaluation of welfare in dromedary camels under pastoralism, and it needs to be applied and refined. If it was used in multiple regions and countries, it would give data useful for the development of welfare standards.

The principle of ‘Good Feeding’ takes into account criteria concerning the prevention of prolonged hunger and thirst, ensuring immediate access to a suitable diet and fresh water to uphold overall health and vitality. Prolonged hunger and thirst can arise when feed and water are not readily available, inaccessible, or fail to meet not only the nutritional needs but also the behavioral needs of grazing and browsing (27). The present protocol thus included indicators of effective availability of feed and water (Animal level) and feeding strategies such as the provision of dromedary camels with the possibility of grazing (Caretaker-Herd level) and to cover their needs using supplementation in case of drought and limited pasture quality. Under natural conditions, camels predominantly engage in grazing and rumination, selecting plants rich in water and minerals (52–54). Mineral salts are crucial for thermoregulation, metabolic health, and water retention in camels (55, 56). Despite gauging feed and water quantity and potential variety, these indicators may overlook nutritional content variations and water quality, both critical factors in preventing health issues (57). Seasonal fluctuations and unpredictable environmental conditions affecting forage and water accessibility and quality may not be fully captured, and individual dietary variations based on age, sex, physiological status, and functionality may not be addressed. Hence, the indicator ‘Body Condition Score’ (BCS), including at the ‘Animal level’, is vital for assessing the long-term welfare conditions of camels. BCS is a robust animal-based indicator for evaluating medium to long-term good feeding practices in livestock species, including camels (43, 58, 59). Because of the subjective nature of body condition scoring, standardized training for scorers is highly recommended to ensure consistency (60). Moreover, it is always to take into account that a low BCS could also be associated with health issues (9), and further research inquiries are encouraged to discern the welfare implications associated with each body condition scoring category in dromedary camels, considering factors such as age, physiological state, and rearing purpose. The bucket test, included in the previous available protocol for camel welfare assessment at intensive and semi-intensive systems (8), was considered to be not feasible in the present protocol due to practical constraints (i.e., lack of materials for the implementation of the test in pastoralist settings). Similarly, other reliable ABM, as the capillary refilling time was considered not feasible as many of the camels under pastoralism are unhandled and to evaluate the CRT the animals would have needed a containment. Another indicator, namely the sunken eyes proposed by Abdalla et al. (61) was excluded as it was considered not specific, as this is often present in dehydration status caused by health problems in dromedary camels (44). However, it would be good to find a better indicator of prolonged thirst/dehydration using non-invasive smart technologies.

The principle of ‘Good Housing’ encompasses criteria associated with herd structure and the environment where the animals are experiencing, namely comfort around resting, thermal comfort, and ease of movement. In extensive contexts, where camels exhibit a strong attachment to specific sleeping sites (30), access to suitable resting places overnight significantly contributes to their overall welfare. This provision helps reduce stress and ensure thermal comfort when sheltered areas do exist in the resting environment. Beyond these physical benefits, a resting area facilitates behavioral observations, enabling caretakers to monitor behavior and identify potential signs of distress, pain, or disease. Fenced resting areas also protect against predators, although pastoralists mostly engage in strategic grazing activities and constant surveillance to mitigate potential losses from predators (31, 62). Shaded areas accessible to camels during the day are further beneficial for thermal comfort in high-temperature environments, preventing heat stress (63, 64). Despite camels’ adaptations to extreme temperatures, prolonged heat stress can result in decreased appetite, reluctance to rise, lethargy, and even death. Predisposing factors, including parasitism, lameness, weaning, inadequate nutrition, or obesity, have been listed as risk factors for heat stress (64). Providing the camels with a sheltered and fenced place with access to fresh water and food during the night, would not only enhance camel welfare but also help in the milking practices and the commercialization of the milk. Group size was also inserted as an indicator of good housing, as it significantly influences the general comfort of animals in terms of housing conditions. Where overnight resting areas or daytime shaded areas are small in relative proportion to the group size, not all animals will have free and adequate access to them. Indeed, limited space allowance can lead to a reduction of lying time in animals (65). In addition, overcrowding due to large group size can impair access to resting areas and feed resources, potentially resulting in aggressive interactions and increased risk of injury and distress (66). A group size larger than 30 animals, which doubles the average group size in natural populations (8–15 camels) (30), is also associated with a significantly increased risk of health issues (29). The latter is the reason for this threshold within the present protocol. However, as group size is largely dependent on managerial decisions, and it is the same for all the assessed herds, this specific indicator was included as a Good housing indicator at the Caretaker-Herd level.

Good Housing at the Animal level encompassed several indicators. To evaluate the impact of inadequate environment on resting and walking spaces in dromedary camel welfare (67), three specific indicators were included, namely risk of injury, presence of ectoparasites, and camel coat cleanliness. Risk of injury is a critical indicator in identifying potential hazards in the environment (e.g., the presence of sharp and protruding elements, damaged fencing areas, or elements used for animal handling/restraining, but also rubbish), then in preventing injuries and the ingestion of foreign bodies and promoting a safe living space for dromedaries. On the other hand, the presence of ectoparasites (e.g., ticks) and the cleanliness of camel coats are indicators that address the specific impact of the hygiene conditions of the living areas on the overall health and well-being of dromedary camels (63). Moreover, as good housing also responds to the guarantee of freedom of movement, some indicators on the restrain methods (e.g., presence of hobbles, restrained in three legs) were included. Movement control in camels is reported to influence metabolism, benefiting feed digestion and nutrient absorption, thus the overall welfare and performance of camels (68). In pastoralist herds, the majority of camels can move freely for many hours a day. However, a small percentage may experience restricted movement on account of various factors based on individual health considerations and reproductive status (13, 69). For instance, some animals may need partial restraining due to illness, injury, or the necessity for specialized care. Additionally, limiting the movement of selected animals can aid in the strategic management of breeding programs, allowing for controlled interactions and monitoring. Although restraining can be beneficial in some instances, excessive restrictions lead to stress and discomfort due to the inhibition of movement and expression of natural behavior. Therefore, evaluating the ease of movement at the ‘Animal level’ allows for a focused examination of the welfare of the animals with restricted mobility within a herd.

Concerning the welfare principle of ‘Good Health’, the indicators included at the Caretaker-Herd level aimed to address the presence of injury, disease, pain, and pain induced by management procedures within nomadic pastoralist contexts. The inefficacy of traditional treatments, the absence of professional surgical interventions, and improper use of veterinary drugs have been listed as causes of mortality and calf mortality has been identified as one of the major welfare concerns under this type of management (13, 70). In fact, traditional husbandry practices and calf mortality rates continue to be among the major constraints affecting camel overall production in extensive pastoral settings (71). The present protocol therefore introduced indicators to scrutinize camel health care and management, particularly examining the regular monitoring of herd health (preventive and curative healthcare measures) and the expertise dedicated to camel well-being (i.e., routine involvement of veterinarians) and 1-year-old calf mortality. Concerning calf mortality in dromedary camels, these indices are reported to be quite variable (5.1–50%) due to the complex interplay of biological, environmental, and human-related factors that vary across different locations and contexts (32–36, 38, 39). Following the recommendations of Nagy et al. (38) and in virtue of the potential application of a Food Safety Management System (FSMS) to extensive livestock farming, we suggested a threshold of 10% for calf mortality rates to accurately assess the welfare of dromedary camel pastoralist herds. However, in comparison with other species, the rate is high and it could be used as an iceberg indicator (25). In addition, the absence of formal recording is considered the worst condition in terms of animal welfare within this protocol, as it may make it difficult for the proper implementation of corrective measures. Further indicators addressing animal identification and commingling (mixing camels from different herds or with other livestock species) provide a broader perspective on herd health dynamics. While an identification and traceability program is crucial for individual monitoring and minimizing the risks to public health and welfare (i.e., eradication programs) (37), commingling may expose animals to increased epidemiological risks (72). Besides, the procedures used for animal identification can induce pain; this condition is proposed to be addressed in the current protocol.

Additional indicators to be assessed at the Animal level for the principle of ‘Good Health’ were introduced. They are mainly clinical indicators that can signal the presence of a disease or anatomical irregularity. They are the same as the previous protocol (8) as they are easy to assess also under pastoralist conditions. While comprehensive, this indicator demands a holistic and expert understanding of camel health and the ability to differentiate between normal variations and abnormal conditions. Lastly, the presence of pain-induced management practices (i.e., cauterization, branding, nose peg, and mutilation) (73–75) and evident pain highlight the importance of assessing not only physical health but also the impact of human interventions on the psychological health of camels. Although a composite pain scale has been recently conceived based on the literature available on other mammal species (44), this needs application and validation. This validation would lead to the refinement of the specific animal-based indicator of ‘Evident pain’ included in the present protocol.

The principle of ‘Appropriate Behavior’ encompasses the evaluation of the domain of behavior, considering the possible effects that environment, con-specific, and humans may have on animal behavior (76). Given that camels are herd animals, the assessment of social behavior constitutes a valuable measure of the general welfare of the herd. Positive social camel-camel interactions (i.e., cow-calf contact, allogrooming, and sniffing among others) provide a window into the animals’ social cohesion, indicating positive affiliations that contribute to a harmonious herd structure. Conversely, aggressive camel-camel interactions highlight potential sources of stress or conflict within the herd. Identifying and understanding these aggressive behaviors is crucial for managing social dynamics and preventing injuries. Additionally, as reported by Padalino and collaborators (77), the manifestation of locomotor and oral stereotypies in camels is heightened by factors like inadequate living conditions. In extensive systems, moreover, the adequacy of time spent grazing and rumination might suggest a favorable welfare state, considering that in natural ecosystems these animals spend the majority of the time grazing and ruminating (78). In fact, feeding and rumination behavior might be related to the size of the group and the feeding/housing areas. Specifically, larger group sizes in relatively reduced areas could be expected to increase alert behavior and individual vigilance (i.e., aggressive behavior is more prevalent due to increased social interactions in reduced areas), and decrease eating time (65, 66, 79). The human-camel relationship can be evaluated using the approaching test previously applied in camels kept in intensive and semi-intensive conditions (8). The presented list of indicators could be further implemented in the near future including other behavioral traits, such as the Qualitative Behavioral Assessment (QBA), as in other species, after some specific validation studies on the term to use to evaluate camel affective states, both positive (e.g., calm, content) and negative (e.g., agitated, frustrated) emotional states (80) will be conducted.

Four indicators under the ‘Appropriate Behavior’ principle at the ‘Caretaker-Herd level’ were also included. One of the indicators focused on the presence of aggressive or dangerous animals, as aggressivity can be abnormal behavior led by inappropriate management. Moreover, it is well known that the presence of aggressive animals within a herd may impair the welfare of the other herd members, but it may be triggered by stressful situations and therefore not always observable in a short time window (81). As dromedary males can exhibit aggression during the breeding season (68), an intermediate score for this situation was created. The second indicator regarded the years of experience that caretakers have in handling camels, as lack of experience is a well-known welfare hazard (25). The third indicator focused on the ratio between the number of caretakers and the number of camels kept in the herd. The caretaker-to-animal ratio emerges as a critical factor influencing animal welfare, particularly linked to animals’ responses to humans or the quality of human-animal relationships. As a reference, Des Roches (50) found that the proportion of cows that accepted being touched increased with the worker/cow ratio on the farm and the caretaker’s years of experience. This phenomenon may be attributed to cows becoming more familiar with interacting with individuals exhibiting diverse appearances and gestures through the process of stimulus generalization. The fourth indicator focuses on caretaker attitudes during animal handling (40, 41). The OIE Terrestrial Animal Health Code (42) emphasizes the caretaker’s responsibility for humane animal handling and care, necessitating adequate skills and knowledge to ensure adherence to animal welfare principles. Within the framework of human-animal interactions, the caretaker’s attitudes depict the frequency and nature of engagements between the herd manager and the animals (82). Therefore, on-field, objective evaluation of caretaker’s attitudes in camel handling, along with caretaker-to-animal ratio and behavioral tests proposed at the ‘Animal level’ (i.e., approaching test), is crucial for a comprehensive understanding of the human-animal relationship.

Finally, concerning the aggregation of measures from indicators to construct compound welfare indices, a model was adapted to be applied through the present protocol, following the methodology and conclusions by Menchetti et al. (9). However, based on the results of the applicability of the whole scoring and aggregation system, PAIs (indices that provide scores for each welfare principle) are concluded to be more useful and effective than LAIs (indices that provide scores for each evaluation level, i.e., Caretaker-Herd and Animal). At a practical level, PAIs could be used to identify the major issues constraining camel welfare, thus suggesting preventive, mitigating, and corrective actions to the animal caretakers (9). Therefore, LAIs were not included in the current protocol. The classifications of the herds based on the PAIs and the traffic light systems are in line with the literature, and data collection is required to see if the proposed thresholds can be applicable under pastoralism conditions. As reported in the literature (8–11), the findings of a welfare assessment should be interpreted as a snapshot influenced by the particular season and climatic conditions, and useful to suggest best practices to apply in a particular principle/domain and decide when to re-assess the herd to see if the welfare of the animals have improved.

This is a theoretical protocol and it has all the limitations already listed for similar papers (51, 76). In particular, the limited literature related to dromedary camel welfare forced the authors to look into the welfare assessment of other species kept under extensive husbandry systems, and this may require a refinement of the protocol after it is applied more times. Moreover, for the moment this protocol has been developed for adult dromedary camels, in any physiological state (pregnant and non-pregnant, lactating and non-lactating), a specific protocol for calves and pre-puberty animals should be developed, applied, and validated as it was done in other species (10). In the literature, welfare assessment protocols are tailored based on the age (young vs. adult animals) and the husbandry systems (intensive vs. extensive husbandry systems), more specific protocols based on seasons or physiological states could be more accurate but would require further studies. Data collections are also needed to refine and validate the scoring systems and the suggested PAIs and classifications. Currently, there is a scientific debate whether aggregation should be performed and how it should be performed, and how often herds should be assessed to safeguard animal welfare during the entire life of the animals (10, 11). Finally, a level of investigation is based on an interview with the farmer, and the answers could be biased by the farmer’s background, education, and experience (49). However, notwithstanding the aforementioned limitations and the fact that the protocol needs future applications and refinements, this is a first step; the current study indeed proposes a tool that has the benefit of using a standardized protocol in animal welfare assessment (42). However, it is worth highlighting that the introduced protocol is not intended solely for research purposes but to encourage governmental organizations and expert advisors to start assessing the welfare of camels with a standardized protocol, as only when there will be a harmonized way in welfare data collection, there may be enough data and research-based evidence to propose welfare standards for camels.

## 5 Conclusion

This theoretical paper describes the process of how a new tool to assess welfare in dromedary camels kept under nomadic conditions was developed. Several indicators were selected at two levels of investigations based on literature and expert knowledge and the reasons beyond their selection and scores and their limitations were discussed. However, the presented protocol signifies the initial phase of an extensive process starting with its application in different regions and countries, the refinement and validation of the proposed indicators, and the identification of thresholds for their acceptability. Further studies are therefore needed to apply the present protocol on several herds kept under pastoralism; data will be firstly collected on a high number of animals (at least 1,000) kept in different herds (at least 50) in a specific area of a country, and then similar data collections will be carried out in different countries until there will be data enough to propose minimal welfare standards. The protocol could also be refined and applied to assess the welfare of Bactrian camels under pastoralism, but more studies should be carried out to test its feasibility.

## Data availability statement

The original contributions presented in the study are included in the article/supplementary material, further inquiries can be directed to the corresponding authors.

## Author contributions

BP: Writing – original draft, Resources, Funding acquisition, Data curation, Conceptualization. LM: Writing – review & editing, Data curation, Conceptualization.
